# Nonresponse of basal cell carcinomas to immune checkpoint inhibition therapy for melanoma: A case series

**DOI:** 10.1016/j.jdcr.2026.01.049

**Published:** 2026-02-06

**Authors:** Elizabeth F. Sher, Ata S. Moshiri, Ian W. Tattersall

**Affiliations:** a The Ronald O. Perelman Department of Dermatology, New York University Grossman School of Medicine, New York, New York; b Perlmutter Cancer Center, New York University Langone Health, New York, New York

**Keywords:** basal cell carcinoma, case series, immune checkpoint blockade, medical dermatology, oncology

Immune checkpoint inhibition has revolutionized the treatment of numerous advanced malignancies.^1^ For melanoma alone, the 5-year overall survival for patients with metastatic disease has changed from less than 10% to 60% with the introduction of immune checkpoint inhibitors (ICIs).^1^^,^^2^ Basal cell carcinoma (BCC) is the most frequently diagnosed malignancy in humans, and its rates continue to rise in incidence due to population aging and increased exposure to ultraviolet radiation.^3^ In the United States alone, millions of cases are diagnosed annually, and the number of procedures performed for BCC and other keratinocyte carcinomas far exceeds those for all other cancers combined, resulting in a major public health and economic challenge.^4^^,^^5^ Although BCC rarely metastasizes and mortality is low, it can cause significant morbidity, particularly in cosmetically and functionally sensitive areas like the head and neck, which leads to disfigurement and impairment.^5^

The FDA recently approved the use of ICIs for treatment of locally advanced and metastatic BCC.^6^ Given the high tumor mutational burden characteristic of many BCCs, these tumors are theoretically good candidates for immunotherapy.^7^ Unfortunately, despite promising preliminary evidence for immunotherapy usage in advanced BCC cases, PD-1 inhibitors are effective only in a small subset of patients.^8^ Furthermore, predictive biomarkers of response to immune checkpoint blockade have not been identified.^9^ These challenges raise an important question: might immunotherapy, when administered for other primary indications, also confer incidental benefit against nonadvanced BCC?

Here we describe 4 patients who were diagnosed with primary BCC during treatment of metastatic melanoma, and whose BCCs did not respond to treatment with anti-PD-1 therapies. It is notable that the BCCs we report were all either large (>2 cm) or exhibited aggressive histology. Our findings suggest that, although ICI therapy can treat advanced or metastatic BCCs, it should not be assumed that immunotherapy administered for other indications will be sufficient to treat newly identified primary BCCs.

## Case 1

**Fig 1:**
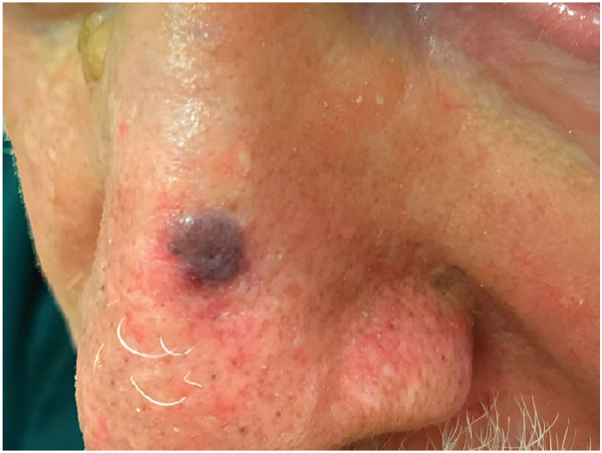
Basal cell carcinoma case 1. Telangiectatic *purple papule* on the bridge of the nose.

**Fig 2:**
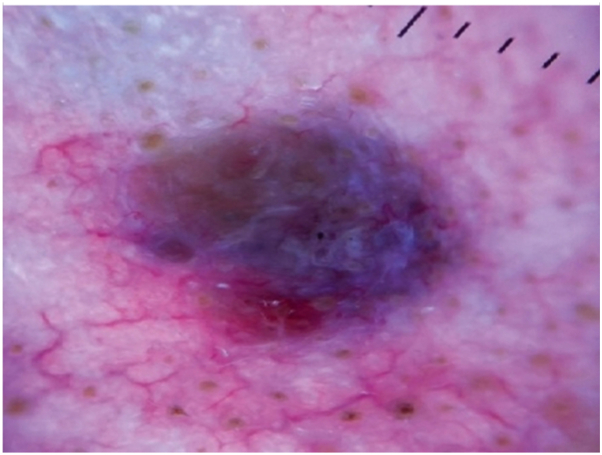
Basal cell carcinoma case 1. Dermoscopy for case 1 reveals *large blue-gray ovoid* nests, arborizing vessels and an area of excoriation along the inferior border of the nodule.

**Fig 3:**
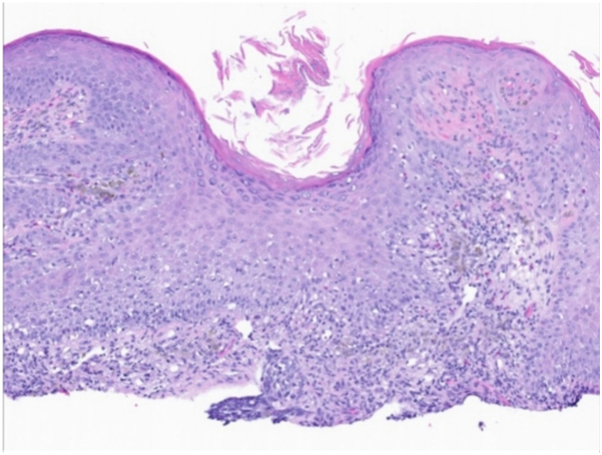
Basal cell carcinoma case 1. Pathology for case 1; Higher power magnification (100×) highlights jagged, infiltrative basaloid nodules with stromal retraction seen best at the base of the specimen.

A 91-year-old man presented to his dermatologist with a 2.1 cm × 1.9 cm blue-black-brown nodule with regression on his left shoulder for 4 months, along with multiple blue-black-brown satellite nodules on the head and chest. Biopsy and subsequent evaluation revealed stage IV (pT0pN0pM1a) melanoma with 9 observed cutaneous satellite lesions on head and chest. At the time, a skin exam performed by dermatology revealed no other lesions concerning for cancer. The patient was treated with pembrolizumab for 1 year, with post-treatment biopsy showing tumor melanosis and no viable tumor. A month after his last dose, he was referred to dermatology by his oncologist for evaluation of a newly appreciated 1 cm purple, telangiectatic papule (Figs 1 and 2) on the nasal bridge. Biopsy confirmed an infiltrative BCC (Fig 3), which was treated by Mohs micrographic surgery.

## Case 2

A 71-year-old woman was diagnosed with a stage IIC (pT4bpN0cM0) 5.1 mm spindle cell melanoma on the left upper back. The patient underwent a wide local excision and sentinel lymph node biopsies, which were negative for melanoma. A week before starting immunotherapy, she presented with a bleeding mamillated pearly pink 3 by 2 cm plaque with rolled borders and overlying coarse scale on the left back (Supplementary Fig 1 and 2, available via Mendeley at https://data.mendeley.com/datasets/6r6zyjd986/1). Biopsy confirmed BCC with nodular and superficial patterns (Supplementary Fig 3, available via Mendeley at https://data.mendeley.com/datasets/6r6zyjd986/1). She was scheduled to begin adjuvant pembrolizumab therapy, so surgical management of the BCC was deferred based on the possibility that the lesion might demonstrate a therapeutic response to immunotherapy. However, 3 months into pembrolizumab therapy, there was no clinical response of the BCC, and the lesion was treated by surgical excision. The patient went on to develop additional BCCs after completing immunotherapy but has had no recurrence of her melanoma.

## Case 3

A 77-year-old man presented with a growing, irregular, brown pigmented lesion involving the left lower eyelid and infraorbital cheek. Biopsy showed stage IIA (cT2b, cN0, cM0) 1.9 mm invasive melanoma with ulceration and 3 mitoses/mm^2^. Due to concerns over disfigurement from surgery, he received 4 months of neoadjuvant pembrolizumab and ipilimumab. Two months later, biopsy revealed only tumoral melanosis with no viable tumor. At that visit, no other concerning lesions were noted. One year later, he developed metastatic disease with peritoneal involvement and liver ascites and was treated with ipilimumab and nivolumab for 2 months, followed by maintenance nivolumab for 5 months, then nivolumab and relatlimab (a LAG-3 inhibitor) for 15 months. Five months into his maintenance nivolumab treatment, the patient presented to dermatology with a 2 cm eroded pink nodule on the right lateral back (Supplementary Fig 4 and 5, available via Mendeley at https://data.mendeley.com/datasets/6r6zyjd986/1). Biopsy revealed a nodular BCC (Supplementary Fig 6, available via Mendeley at https://data.mendeley.com/datasets/6r6zyjd986/1), which was monitored for 4 months as patient continued immunotherapy. The lesion persisted despite continued, and after 4 months it was treated with electrodesiccation and curettage.

## Case 4

**Fig 4:**
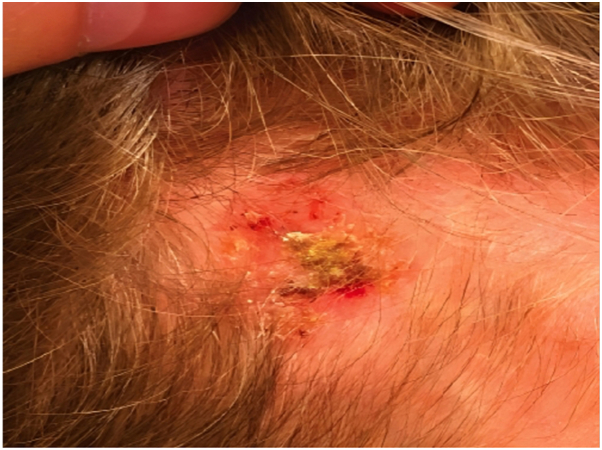
Basal cell carcinoma case 4. Crusted *pink pearly plaque*.

**Fig 5:**
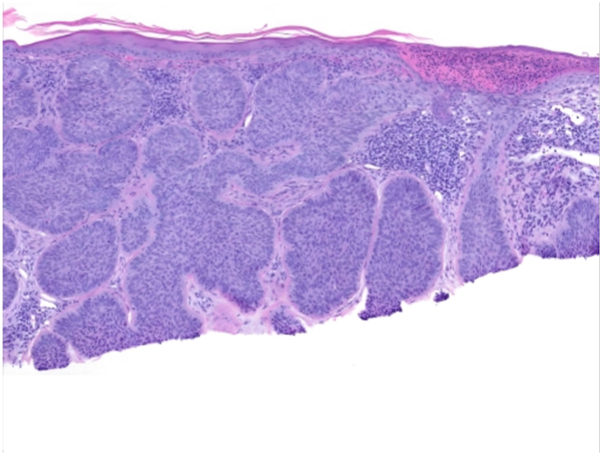
Basal cell carcinoma case 4. Pathology for case 4; Higher power magnification (100×) highlights single cell necrosis and fibromucinous stroma surrounding the tumor nodules with retraction seen best at the base of the specimen.

A 61-year-old woman was diagnosed with a stage IIIc (pT4apN1acM0) invasive melanoma with 3.3 mm Breslow thickness on her left leg. The patient was treated with wide and deep excision of the primary lesion with sentinel lymphadenectomy followed by nivolumab and pempegaldesleukin (an experimental recombinant interleukin 2) for 3 months, then transitioned to nivolumab maintenance monotherapy. Three months into nivolumab maintenance, a dermatologic skin exam revealed a new 2 cm crusted pink pearly plaque on her right scalp (Fig 4). Biopsy of lesion revealed a pigmented nodular BCC (Fig 5), and the lesion was then successfully treated by Mohs micrographic surgery.

## Discussion

Although ICIs have dramatically changed the course of treatment for patients with advanced malignancies, their effects on the body are wide reaching and incompletely understood.^1^ Given that ICIs are now used to treat both melanoma and nonmelanoma skin cancer, one might theorize that patients on immunotherapy might be protected against the development of new cutaneous malignancies, or that immunotherapy given for the treatment of more aggressive cancers might be able to treat coincident nonmelanoma skin cancer. However, our observations add to a small body of case reports that have described primary BCCs that arise during or persist despite immune checkpoint blockade, demonstrating that the development and progression of nonmelanoma skin cancer remains a problem for patients even during immunotherapy.^10^^,^^11^ The authors of these reports propose that earlier primary malignancies harbor a lower mutational burden, which may reduce sensitivity to ICIs. Therefore, immune checkpoint blockade may be more suited for “late” disease when tumor cells have acquired more genomic aberrations, allowing them to be more readily recognized by the immune system and accordingly more susceptible to immunotherapy.^11^ Other studies have suggested a shift towards a tumor-promoting Th2 immunity as a BCC develops, identifying an increase in anti-inflammatory cytokines Il-4 and Il-10 in advanced and vismodegib-resistant tumors.^12^

The cases herein described suggest that while ICI treatment can treat advanced or metastatic BCCs, immunotherapy administered for other indications does not necessarily also treat coincident BCCs, underscoring the importance of continued skin surveillance and proactive management in patients receiving immunotherapy. Skin exams in 2 out of 4 patients prior to the initiation of immunotherapy (cases 1 and 3) revealed no lesions concerning for cancer, indicating that a subset of these patients may have developed new BCCs while on immunotherapy, or that these lesions grew during immunotherapy to a point where they were more clinically detectable. Case 2, in contrast, described a BCC that was definitively identified prior to immunotherapy initiation, but failed to respond to immunotherapy. The exact timing of onset of the lesion in case 4 is unknown, given its large size, it would be expected to have been present 6 months prior at the initiation of immunotherapy, unless it exhibited uncharacteristically aggressive growth.

Importantly, these findings highlight that BCCs may remain refractory to immune checkpoint blockade, whether they are present at the time of treatment initiation or whether they arise during treatment. Notably, the BCCs in our patients were all either large or of aggressive histology; although this observation should by no means be taken to suggest that immunotherapy treatment of other cancers might predispose to more aggressive non-melanoma skin cancer presentations, this signal merits further study.

This case series focuses on a small number of patients at a single institution, and it is important to resist overgeneralization of these observations. Although the authors have not treated patients with BCCs that did respond to immunotherapy for melanoma, this should not be taken to suggest that all early BCC are guaranteed to be resistant to immunotherapy. Systematic studies with larger cohorts will be required to determine whether these therapies influence BCC incidence, progression or clinical behavior.

Going forward, investigation into the genomic alterations of immunotherapy-sensitive vs immunotherapy-resistant BCCs may lead to the identification of new biomarkers for predicting response to immunotherapy in BCCs. More specifically, immunohistochemistry of biopsied BCCs arising in the setting of immune checkpoint inhibition may be valuable to determine the genomic and immune alterations that contribute to ICI resistance in primary lesions. Understanding the immune microenvironment in both naive and advanced BCC may allow for more precise immunotherapeutic modalities as well as indications for how to overcome treatment resistance.

## Conflicts of interest

None disclosed.
